# Clinical effects of Shou-Wu Jiang-Qi Decoction combined acupuncture on the treatment of Polycystic Ovarian Syndrome with kidney deficiency, phlegm and blood stasisness: Study protocol clinical trial (SPIRIT Compliant)

**DOI:** 10.1097/MD.0000000000019045

**Published:** 2020-03-20

**Authors:** Wenting Xu, Mengyu Tang, Jiahui Wang, Lihong Wang

**Affiliations:** Department of Reproduction, Zhangjiagang TCM Hospital Affiliated to Nanjing University of Chinese Medicine, Zhangjiagang, Suzhou, Jiangsu, China.

**Keywords:** acupuncture, polycystic ovary syndrome, Shouwu Jiangqi Decoction, traditional Chinese medicine

## Abstract

**Background:**

Polycystic ovary syndrome (PCOS) is a female endocrine disease with a high incidence. At present, drug treatment is still the main therapeutic strategy for PCOS. Traditional Chinese medicine has a long history in the treatment of menstrual disorders. Shouwu Jiangqi Decoction (SWJQD) is a traditional herbal medicine prescribed in a clinical setting as a remedy for PCOS. Acupuncture also plays an important role in regulating the menstrual cycle and treating PCOS. This study aims to examine the efficacy and safety of the combination of SWJQD and acupuncture in the treatment of PCOS.

**Methods:**

This randomized controlled trial will be conducted with a total of 81 participants diagnosed with PCOS. The participants will be randomly divided into 3 treatment groups: group A will receive SWJQD combined with acupuncture; group B, SWJQD combined with sham acupuncture; and group C, metformin. Each treatment will last 3 months. The primary outcomes include the Homeostatic Model Assessment of Insulin Resistance (HOMA-IR) index and the Oral Glucose Tolerance Test. The secondary outcome measures include sex hormone levels, body mass index, ovulation rate, clinical pregnancy rate, and complete genome sequencing data. Adverse events will be recorded during the intervention and follow-up.

**Results:**

This study will investigate whether the combination of SWJQD and acupuncture can alleviate the clinical symptoms and improve insulin resistance in patients with PCOS. The results of this study are expected to provide clinical evidence for the application of the combination of SWJQD and acupuncture in patients with PCOS.

**Trial registration:**

Chinese Clinical Trial Registry: ChiCTR1900028106, ChiMCT1900002826 (registered on December 12, 2019).

## 1 Introduction

Polycystic ovary syndrome (PCOS) is a common female reproductive disease that mainly occurs in adolescents and at a childbearing age.^[1]^ The clinical manifestations are persistent anovulation, scanty ovulation, insulin resistance (IR), excessive androgen secretion and polycystic changes in the ovary, leading to irregular menstruation and infertility.^[2]^ The pathogenesis of PCOS is still unclear. It is currently believed that it may be related to genetic, environmental, or mental health factors, as well as other comprehensive factors.^[3–5]^

Epidemiological studies have confirmed that PCOS is a serious gynecological disease with a high incidence in China and across the globe.^[6,7]^ PCOS patients have high risk of type 2 diabetes, obesity, hypertension, metabolic syndrome, and cardiovascular disease.^[8]^ Due to the endocrine and metabolic nature of the disorder, PCOS patients may have some difficulty with fertility, as well as be experiencing severe physical and psychological distress.^[9,10]^

Recent studies have revealed that inflammation and obesity are important factors in the pathophysiological basis of PCOS, while both hyperandrogenemia and IR have established key roles in its development.^[4,11]^ In 1980, Burghen et al first proposed that IR is a major clinical characteristic of PCOS.^[12]^ In the next several decades, several researches have confirmed that there is a close relationship between PCOS and IR.^[13]^ Any abnormality in the insulin signaling pathway can lead to IR. The mechanism of IR is related to disordered post-insulin receptor signal transduction, particularly involving the phosphatidylinositol trikinase signaling pathway.^[4]^

At present, the main aim of the routine treatment of PCOS is to improve IR, reduce hyperandrogenemia and thus induce ovulation as the main therapeutic target by lifestyle and drug intervention.^[14]^ Improving endocrine function and inducing ovulation is an important way to improve the pregnancy rate among infertile patients with PCOS.^[15,16]^ Surgical and assisted reproductive techniques are used as second-line therapies to assist the pregnancy.^[17]^

Studies have found that acupuncture can regulate the reproductive endocrine function of PCOS patients through multiple signaling pathways and targets in the hypothalamus-pituitary-gonadal/adrenal axis,^[18]^ thus treating PCOS and improving local symptoms such as hairy acne.^[19,20]^

Traditional Chinese medicine also has certain advantages in regulating female endocrine function, hormone level and menstruation.^[18,21]^ Animal research has confirmed that Shouwu Jiangqi Decoction (SWJQD) has potent therapeutic effects in the rat model PCOS-IR by correcting defective insulin signaling transduction.^[22]^

Therefore, in this study we will try to combine the advantages of traditional Chinese medicine and acupuncture in the treatment of PCOS. To achieve this, we will carry out a standardized clinical trial to improve the treatment plan and evaluation criteria, and provide an objective theoretical basis and methodology for the treatment of PCOS by acupuncture combined with SWJQD.

## 2 Methods and study design

### 2.1 Objective

The purpose of this study is to assess the clinical efficacy of SWJQD combined with acupuncture in the treatment of PCOS.

### 2.2 Overall study design

**Figure 1:**
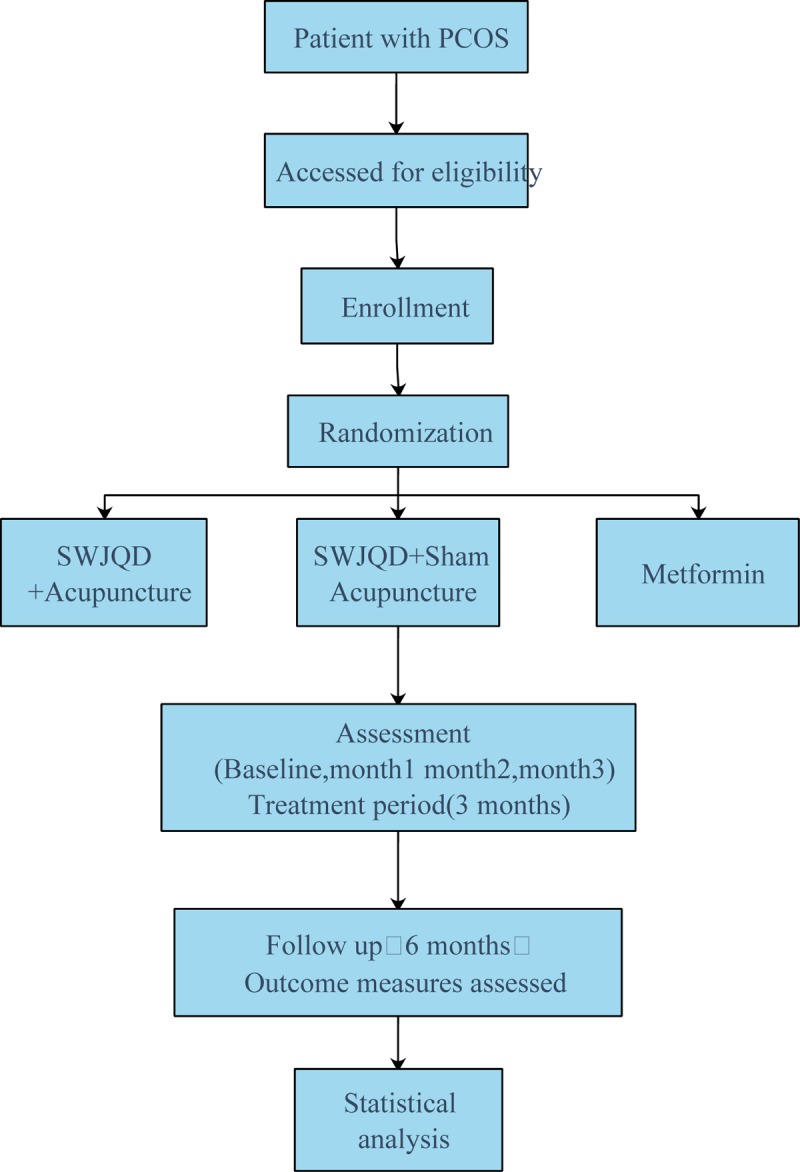
Flowchart of the study procedure.

This is a randomized controlled trial to investigate the clinical effect of SWJQD combined with acupuncture in improving the insulin sensitivity and regulation of sex hormones in Chinese women with PCOS compared with metformin. The study period will include 3 months of pharmacological interventions, acupuncture and a follow-up visit. The general design of this study is demonstrated in Figure 1 and Table 1.

**Table 1:**
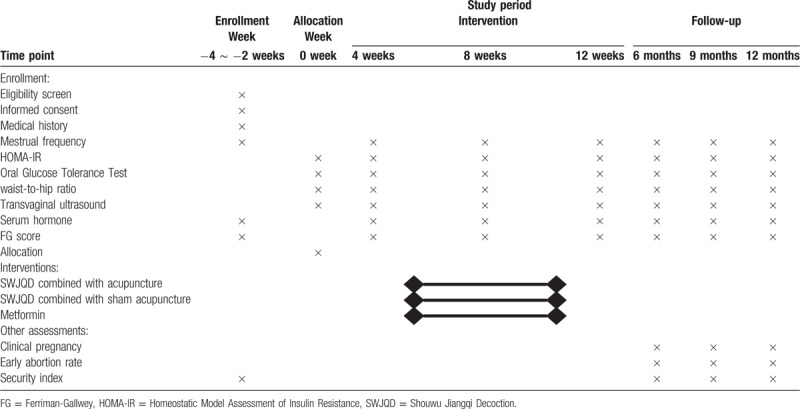
Schedule of enrollment, intervention, and assessment.

### 2.3 Study setting

Chinese women diagnosed with PCOS will be enrolled at the Department of Reproduction, Zhangjiagang TCM Hospital Affiliated to Nanjing University of Chinese Medicine, China. The recruitment will begin in January 2020 and is expected to be completed in March 2022.

### 2.4 Inclusion criteria

The inclusion criteria for the participants are as follows:

(1) Chinese women aged 18 to 35 years.
(2) Body mass index (BMI) between 24 to 32 kg/m^2^.^[23,24]^
(3) Traditional Chinese medicine syndrome differentiation: kidney deficiency, phlegm and blood stasisness.
(4) Satisfying the Rotterdam PCOS diagnostic criteria in adults, including at least 2 of the 3 biochemical or clinical hyperandrogenism characteristics^[25,26]^: (a) Irregular cycles and ovulatory dysfunction (an intermenstrual interval >35 or <21 days or <8 cycles per year) or amenorrhea (>90 days for any 1 cycle), (b) clinical and/or hyperandrogenism: Clinical hyperandrogenism on the Chinese Mainland: Ferriman-Gallwey (FG) score ≥5^[27,28]^; biochemical hyperandrogenism: total testosterone (T) >2.6 nmol/l and free testosterone ≥6.0 pg/ml,^[29]^ (c) polycystic ovarian morphology (the threshold for PCOM should be on either ovary, a follicle number per ovary of >20 and/or an ovarian volume ≥10 ml, ensuring no corpora lutea, cysts or dominant follicles are present.
(5) Presence of IR. The criteria of IR is defined by the homeostatic model assessment (HOMA-IR) index ([fasting serum insulin (μU/ml) × fasting blood glucose (mmol/l)] / 22.5). A value ≥2.14 is considered to be indicative of IR.^[30]^
(6) No birth planning in the future 3 months.
(7) Being able to follow the plan of treatment, examination, and follow-up.
(8) Being able to fully understand and voluntarily sign informed consent.

### 2.5 Exclusion criteria

The exclusion criteria for the participants consist of:

(1) Presence of abnormal liver and kidney functions
(2) Presence of any other endocrine disorders such as:
  (a) uncorrected thyroid disease
  (b) type 1 or 2 diabetes mellitus or hypertension
  (c) Cushing's syndrome
  (d) Congenital adrenal hyperplasia
  (e) Suspected androgen-secreting adrenal or ovarian tumor.
(3) History of alcoholism, malignant tumors, epilepsy, or mental disorders.
(4) History of coronary heart disease, severe arrhythmia, cardiopulmonary insufficiency, or cardiac stenting procedure.
(5) Use of a pacemaker, hearing aid, or any other device with electronic or metal components.
(6) Presence of infectious diseases and severe skin lesions.
(7) Pregnant or lactating state.
(8) Unable to cooperate with the research caused by other diseases or reasons;
(9) Use of hormonal or other medication including Chinese herbal prescriptions, which may affect the outcome of the last 2 months.
(10) History of receiving acupuncture treatment in the past 2 months.

### 2.6 Interventions

After baseline measurements, patients satisfying the inclusion criteria will be randomly divided into 1 of the 3 treatment groups, as shown below:

1. Group A: SWJQD combined with acupuncture for 3 months.
2. Group B: SWJQD combined with sham acupuncture for 3 months.
3. Group C: Metformin at a dose of 500 mg 3 times/day for 3 months.

All the participants will be instructed to return to visit the doctor every week.

#### 2.6.1 Shouwu Jiangqi Decoction

The SWJQD consists of 10 g of Fallopia multiflora, 12 g of Radix Puerariae, 15 g of Batryticated silkworm, 15 g of Astragalus propinquus Schischkin, 15 g of Dioscorea oppositifolia L., 10 g of Euonymus alatus, 10 g of Cyperus rotundus L., and 10 g of Cuscutae Semen. The traditional Chinese medicine doctor will prescribe the formula according to the clinical symptoms of the participants. Each decoction should be taken twice a day, except during the menstrual period. The SWJQD intervention will last for 3 months.

#### 2.6.2 Acupuncture

The acupoint selection for the acupuncture group will be as follows: Zhongji (RN3), Guanyun (RN4), Zigong (EX-CA1), Sanyinjiao (SP6), Zhongwan (RN12), Tianshu (ST25), Fenglong (ST40), Zusanli (ST36), and Qihai (RN6). Needles (40 mm in length, 0.3 mm in diameter; Hwato Brand, Suzhou Medical Appliance Factory, China) will be inserted into all acupoints (to a depth of 25–30 mm). Following lifting, thrusting, and stimulating until ’de qi’, the needles will be left in the acupoints for 30 minutes before removal. This acupuncture intervention will be conducted for 3 months, except during the menstrual period.

#### 2.6.3 Sham acupuncture

The acupoint selection for sham acupuncture will be: sham Zhongji (RN3), Guanyun (RN4), Zigong (EX-CA1), Sanyinjiao (SP6), Zhongwan (RN12), Tianshu (ST25), Fenglong (ST40), Zusanli (ST36), and Qihai (RN6), which are 1 cun outward to Zhongji (RN3), Guanyun (RN4), Zigong (EX-CA1), Sanyinjiao (SP6), Zhongwan (RN12), Tianshu (ST25), Fenglong (ST40), Zusanli (ST36), and Qihai (RN6), respectively. Needles (15 × 0.3 mm) will be inserted to 2 to 3 mm at the sham acupoints. The sham acupuncture intervention duration will be the same as that for the acupuncture group.

#### 2.6.4 Metformin

In order to reduce the gastrointestinal effects of metformin, the oral dose will be increased gradually as 500 mg per day during the first week, 1000 mg per day during the second week, and 1500 mg per day in the third week and will be maintained at the dose of 500 mg 3 times daily (at morning, noon, and night) from the fourth week onwards, for a total of 3 months.

### 2.7 Outcome measures

#### 2.7.1 Primary outcomes

The clinical effectiveness of 3 months of (A) SWJQD combined with acupuncture, (B) SWJQD combined with sham acupuncture, and (C) metformin for the improvement of IR will be assessed and determined by the HOMA-IR index (calculated from fasting glucose and serum insulin) and the Oral Glucose Tolerance Test.

#### 2.7.2 Secondary outcomes

(1) Sex hormone profile, including estradiol, luteinizing hormone, follicle-stimulating hormone, prolactin, testosterone, progesterone, dehydroandrosterone, and sex hormone binding globulin levels measured on the 2nd to 3rd day of menstruation, before and after the intervention.
(2) BMI (weight/(height)^2^); waist-to-hip ratio (waist circumference/hip circumference), and FG score measured before and after the intervention.
(3) Ovulation rate in every menstrual period.
(4) Clinical pregnancy rate, and early abortion rate in the 9 months follow-up.
(5) Complete genome sequencing of each group before and after the treatment.
(6) Security index tests, including routine blood, liver function, kidney function, and blood lipid tests respectively before and after the intervention.
(7) Investigators will contact subjects weekly by WeChat message or phone call to record the adverse reactions and side effects in this trial.

### 2.8 Sample size calculation

The most important outcome in this study is fasting serum insulin. Sample size was estimated based on the above primary outcome measures. The target sample size calculated by PASS 11.0 is a total of 63 participants (21 participants in the Group A, 21 participants in the Group B, 21 participants in the Group C). This result was based on a power calculation using a 5% significance level, 90% power, and current knowledge of the average serum insulin level. By enrolling 27 participants in each group, we can allow a drop-out rate of 20%.

### 2.9 Recruitment

Eligible subjects will be offered a consent form including information on the background, purpose, possible benefits, and harms of the study after detailed consultation and explanation of the study design. Depending upon the provision of consent, patients will be enrolled.

### 2.10 Randomization and allocation concealment

Eligible participants will be allocated to the three groups in a 1:1:1 ratio by an investigator according to a random sequence generated by a computer. The random number assignment will be generated by an independent statistician who will not be a member of the research team. The random number will be placed in sealed, opaque envelopes and assigned by a staff, who will not be a member of the clinical trial team and participating in data collection. When new participants are enrolled in the study, the doctor will open the envelopes in sequence and assign participants to the corresponding intervention accordingly. All processes will be recorded and saved as appropriate.

### 2.11 Blinding

In this study, patients, research staff, and data statisticians will be blinded to the assignment. However, acupuncture therapists cannot be masked as sham acupuncture will be practiced differently from real acupuncture. Acupuncture therapists will not be allowed to share any information in terms of patient intervention with other researchers, including the data statistician.

### 2.12 Data collection and management

Case report forms (CRFs) will be used to document detailed information related to the study, such as the identity of the participants, clinical information, and any other data collected for the study. The medical records will be kept strictly confidential, input and stored in a password-protected electronic database. The database data and source documents will be checked by a member of the study team not involved in the data collection. The hard copies of all CRFs and clinical data will be stored in a fixed locker for the study team only.

### 2.13 Statistical methods

The data of all participants will be involved in the final statistical analysis according to the intent-to-treat principle. The data statisticians will be blinded to the group allocation. All statistical analyses will be performed in SPSS software v19.0 and a *P* value <.05 will be considered statistically significant.

The continuous variables, such as participants’ biochemical and hormonal profiles including estradiol, luteinizing hormone, follicle-stimulating hormone, prolactin, testosterone, progesterone, dehydroandrosterone, sex hormone binding globulin, body mass index, waist-to-hip ratio, FG score, ovulation rate, clinical pregnancy rate, and early abortion rate will be presented as mean ± SD. Between-group comparisons will be carried out on changes from baseline to after treatment using ANOVA or its non-parametric equivalents. Group comparisons will be carried out using the Kruskal-Wallis test followed by Mann-Whitney *U* test.

### 2.14 Ethics approval

The present clinical trial will be conducted in accordance with the principles of the Declaration of Helsinki and has been approved by the Ethics Committee of Zhangjiagang TCM Hospital (2019–1035). This trial has been registered at www.chictr.org.cn (ChiCTR1900028106, ChiMCT1900002826). The outcomes will mainly use in the clinical evaluation of PCOS. The cost of drug interventions and laboratory tests will all be covered by the study funding. All participants will finally receive a summary of their study data and their personal data will be kept strictly confidential.

### 2.15 Safety assessment

During the clinical trial and follow-up, if any trial-related adverse events occur, the investigator and the clinical trial institution shall ensure that the participant is properly medically treated and truthfully informed of any laboratory abnormalities with clinical significance. Investigators will be vigilant regarding the presence of other diseases in subjects, as well as the possible interference of the combination of drugs in the clinical trial results.

## 3 Discussion

To ensure the quality of this clinical trial, only patients who meet the PCOS inclusion criteria will be recruited. In addition, patients will be excluded if they have diabetes, thyroid disease, high blood pressure or mental illness. Medications related to these diseases may interact with the planned interventions and affect the clinical results.

PCOS is a common reproductive endocrine and metabolic disease that seriously affects patients’ quality of life, fertility, and long-term health.^[31]^ The clinical manifestations of the disease are highly heterogeneous, and the diagnosis and treatment are still controversial. Different treatment methods also exist for the condition. Due to the differences in the age and needs and a high degree of heterogeneity in clinical presentation among PCOS patients, there is a need for individualized treatment measures based on patient complaints, symptoms, treatment needs, and metabolic changes to relieve clinical symptoms, resolve fertility problems, and improve quality of life.

In the past 30 years, Chinese medicine research data illustrate that PCOS is mainly caused by a dysfunctional relationship between the kidney, Chongren, and the palace, and its pathogenesis is closely related to other dysfunctional mechanisms in the liver, kidney, and spleen, with the involvement of phlegm and blood stasis. We designed this study to evaluate the efficacy and safety of acupuncture combined with SWJQD in the treatment of PCOS. The patient's BMI, fasting insulin, blood glucose, menstrual condition, ovulation, and pregnancy status will be recorded and reported.

Acupuncture also has been widely used in China for more than 2500 years. As one of the most important traditional medical techniques in China, acupuncture has certain advantages in treating menstrual disorders and infertility. SWJQD is commonly used in clinical practice, and animal experiments have confirmed that the decoction can effectively improve the IR in rats affected by PCOS. Traditional Chinese medicine and acupuncture have been used as alternative treatment methods in the treatment of PCOS and have been the research hotspots in recent years.^[32]^

Therefore, we considered whether the combination of Chinese medicine and acupuncture could be more effective in improving levels of the relevant hormones in patients with PCOS. There is a lack of relevant clinical evidence in this regard, which we aim to address in this trial. Thus, we will provide supporting evidence for the combination of traditional Chinese medicine and acupuncture to treat PCOS. Many problems have existed in the quality control of the past randomized controlled trials of in the field of traditional Chinese medicine.^[33]^ In order to ensure the quality of our research, avoid bias, and obtain reliable conclusions, we used randomization and blinding methods by using software to randomly generate sequences and conceal random numbers. The limitations of this study are that the sample size is small, and the blinding method cannot be completely used for the acupuncture therapist. Nevertheless, we expect that this study will be able to provide some new evidence regarding traditional Chinese medicine and acupuncture in the treatment of PCOS.

## Author contributions

**Conceptualization:** Wenting Xu, Lihong Wang.

**Investigation:** Mengyu Tang, Jiahui Wang.

**Writing – original draft:** Wenting Xu.

**Writing – review & editing:** Lihong Wang.

Lihong Wang orcid: 0000-0001-7187-5910.

## References

[1] Dumesic DA Oberfield SE Stener-Victorin E Scientific statement on the diagnostic criteria, epidemiology, pathophysiology, and molecular genetics of polycystic Ovary syndrome. Endocr Rev 2015;36:487–525. 26426951 10.1210/er.2015-1018 PMC4591526

[2] Rosenfield RL Ehrmann DA The pathogenesis of polycystic Ovary syndrome (PCOS): the hypothesis of PCOS as functional ovarian hyperandrogenism revisited. Endocr Rev 2016;37:467–520. 27459230 10.1210/er.2015-1104 PMC5045492

[3] Eslamian G Hekmatdoost A Nutrient patterns and risk of polycystic ovary syndrome. J Reprod Infertil 2019;20:161–8. 31423419 PMC6670269

[4] Popovic M Sartorius G Christ-Crain M Chronic low-grade inflammation in polycystic ovary syndrome: is there a (patho)-physiological role for interleukin-1? Semin Immunopathol 2019;41:447–59. 31139895 10.1007/s00281-019-00737-4

[5] Barrea L Marzullo P Muscogiuri G Source and amount of carbohydrate in the diet and inflammation in women with polycystic ovary syndrome. Nutr Res Rev 2018;31:291–301. 30033891 10.1017/S0954422418000136

[6] Ganie MA Vasudevan V Wani IA Epidemiology, pathogenesis, genetics & management of polycystic ovary syndrome in India. Indian J Med Res 2019;150:333–44. 31823915 10.4103/ijmr.IJMR_1937_17 PMC6902362

[7] Qi X Yun C Sun L Gut microbiota-bile acid-interleukin-22 axis orchestrates polycystic ovary syndrome 2019;25:1225–33. 10.1038/s41591-019-0509-0 PMC7376369 31332392

[8] Kakoly NS Khomami MB Joham AE Ethnicity, obesity and the prevalence of impaired glucose tolerance and type 2 diabetes in PCOS: a systematic review and meta-regression. Hum Reprod Update 2018;24:455–67. 29590375 10.1093/humupd/dmy007

[9] Conte F Banting L Teede HJ Mental health and physical activity in women with polycystic ovary syndrome: a brief review. Sports medicine (Auckland, N Z ) 2015;45:497–504. 10.1007/s40279-014-0291-6 PMC4382527 25430602

[10] Balen AH Morley LC Misso M The management of anovulatory infertility in women with polycystic ovary syndrome: an analysis of the evidence to support the development of global WHO guidance. Hum Reprod Update 2016;22:687–708. 27511809 10.1093/humupd/dmw025

[11] Masjedi F Keshtgar S Agah F Association between sex steroids and oxidative status with vitamin D levels in follicular fluid of non-obese PCOS and healthy women. J Reprod Infertil 2019;20:132–42. 31423416 PMC6670262

[12] Burghen GA Givens JR Kitabchi AE Correlation of hyperandrogenism with hyperinsulinism in polycystic ovarian disease. J Clin Endocrinol Metab 1980;50:113–6. 7350174 10.1210/jcem-50-1-113

[13] Moran LJ Norman RJ Teede HJ Metabolic risk in PCOS: phenotype and adiposity impact. Trends Endocrinol Metab 2015;26:136–43. 25591984 10.1016/j.tem.2014.12.003

[14] Pundir J Charles D Sabatini L Overview of systematic reviews of non-pharmacological interventions in women with polycystic ovary syndrome. Hum Reprod Update 2019;25:243–56. 30608609 10.1093/humupd/dmy045

[15] Sharpe A Morley LC Tang T Metformin for ovulation induction (excluding gonadotrophins) in women with polycystic ovary syndrome. Cochrane Database Syst Rev 2019;12:Cd013505. 31845767 10.1002/14651858.CD013505 PMC6915832

[16] Franik S Eltrop SM Kremer JA Aromatase inhibitors (letrozole) for subfertile women with polycystic ovary syndrome. Cochrane Database Syst Rev 2018;5:Cd010287. 29797697 10.1002/14651858.CD010287.pub3 PMC6494577

[17] Lepine S Jo J Metwally M Ovarian surgery for symptom relief in women with polycystic ovary syndrome. Cochrane Database Syst Rev 2017;11:Cd009526. 29125183 10.1002/14651858.CD009526.pub2 PMC6486107

[18] Johansson J Redman L Veldhuis PP Acupuncture for ovulation induction in polycystic ovary syndrome: a randomized controlled trial. Am J Physiol Endocrinol Metab 2013;304:E934–943. 23482444 10.1152/ajpendo.00039.2013 PMC4116535

[19] Lim CED Ng RWC Cheng NCL Acupuncture for polycystic ovarian syndrome. Cochrane Database Syst Rev 2019;7:Cd007689. 31264709 10.1002/14651858.CD007689.pub4 PMC6603768

[20] Chen H Lim CED The efficacy of using acupuncture in managing polycystic ovarian syndrome. Curr Opin Obstet Gynecol 2019;31:428–32. 31567447 10.1097/GCO.0000000000000582

[21] Moini Jazani A Nasimi Doost Azgomi H Nasimi Doost Azgomi A A comprehensive review of clinical studies with herbal medicine on polycystic ovary syndrome (PCOS). DARU 2019;27:863–77. 31741280 10.1007/s40199-019-00312-0 PMC6895349

[22] Wang LH Wang X Yu XZ Potent therapeutic effects of Shouwu Jiangqi decoction on polycystic ovary syndrome with insulin resistance in rats. Chin J Integr Med 2016;22:116–23. 26179926 10.1007/s11655-015-2147-9

[23] Stepto NK Cassar S Joham AE Women with polycystic ovary syndrome have intrinsic insulin resistance on euglycaemic-hyperinsulaemic clamp. Hum Reprod (Oxford, England) 2013;28:777–84. 10.1093/humrep/des463 23315061

[24] Chang RJ Nakamura RM Judd HL Insulin resistance in nonobese patients with polycystic ovarian disease. J Clin Endocrinol Metab 1983;57:356–9. 6223044 10.1210/jcem-57-2-356

[25] Revised 2003 consensus on diagnostic criteria and long-term health risks related to polycystic ovary syndrome. Fertil Steril 2004;81:19–25. 10.1016/j.fertnstert.2003.10.004 14711538

[26] Azziz R Carmina E Dewailly D Positions statement: criteria for defining polycystic ovary syndrome as a predominantly hyperandrogenic syndrome: an Androgen Excess Society guideline. J Clin Endocrinol Metab 2006;91:4237–45. 16940456 10.1210/jc.2006-0178

[27] Hatch R Rosenfield RL Kim MH Hirsutism: implications, etiology, and management. Am J Obstet Gynecol 1981;140:815–30. 7258262 10.1016/0002-9378(81)90746-8

[28] Zhao X Ni R Li L Defining hirsutism in Chinese women: a cross-sectional study. Fertil Steril 2011;96:792–6. 21762890 10.1016/j.fertnstert.2011.06.040

[29] Ni RM Mo Y Chen X Low prevalence of the metabolic syndrome but high occurrence of various metabolic disorders in Chinese women with polycystic ovary syndrome. Eur J Endocrinol 2009;161:411–8. 19542239 10.1530/EJE-09-0298

[30] Chen X Yang D Li L Abnormal glucose tolerance in Chinese women with polycystic ovary syndrome. Hum Reprod 2006;21:2027–32. 16684838 10.1093/humrep/del142

[31] Azziz R Carmina E Chen Z Polycystic ovary syndrome. Nat Rev Dis Primers 2016;2:16057. 27510637 10.1038/nrdp.2016.57

[32] Ong M Peng J Jin X Chinese herbal medicine for the optimal management of polycystic ovary syndrome. Am J Chin Med 2017;45:405–22. 28359195 10.1142/S0192415X17500252

[33] Kwon CY Lee B Park KS Oriental herbal medicine and moxibustion for polycystic ovary syndrome: a meta-analysis. Medicine 2018;97:e12942. 30412108 10.1097/MD.0000000000012942 PMC6221674

